# Impact of relative dose intensity of oxaliplatin in adjuvant therapy among stage III colon cancer patients on early recurrence: a retrospective cohort study

**DOI:** 10.1186/s12885-021-08183-y

**Published:** 2021-05-10

**Authors:** Jolanta Żok, Michał Bieńkowski, Barbara Radecka, Jan Korniluk, Krzysztof Adamowicz, Renata Duchnowska

**Affiliations:** 1 Department of Chemotherapy, Center of Pulmonology and Chemotherapy, 58-580 Szklarska Poręba, Poland; 2 Department of Pathomorphology, Medical University of Gdańsk, 80-214 Gdańsk, Poland; 3 Department of Oncology, Institute of Medical Science, University of Opole, 46-020 Opole, Poland; 4 Department of Oncology, Military Institute of Medicine, 04-141 Warsaw, Poland; 5 Department of Oncology, Regional Oncology Center, 80-210 Gdańsk, Poland

**Keywords:** Colon cancer, Adjuvant chemotherapy, Oxaliplatin, Cumulative dose, Relative dose intensity

## Abstract

**Background:**

Oxaliplatin-based therapy with FOLFOX-4 or CAPOX administered over 6 months remains the standard adjuvant treatment for stage III colon cancer (CC) patients. However, many patients experience dose reduction or early termination of chemotherapy due to oxaliplatin toxicity, which may increase the risk of early recurrence. The objective of this study was to analyze the relationship between the relative dose intensity of oxaliplatin (RDI-O) and early recurrence among stage III CC patients.

**Methods:**

The study included 365 patients treated at five oncology centers in Poland between 2000 and 2014. Survival analysis was performed using the Kaplan-Meier method. Univariate analysis was performed using the Cox proportional hazard model; multivariate analysis was performed with the stepwise forward approach. For all analyses the α level of 0.05 was employed.

**Results:**

The median follow-up was 51.8 months (range 8.2–115.1). Early recurrence < 36 months after surgery occurred in 130 patients (37.8%). In this group 51 (39.2%) and 87 (66.9%) of patients were low and high-risk, respectively. Receipt < 60% of RDI-O was associated with early recurrence within 18 months after surgery (OR = 2.05; 95%CI: 1.18–3.51; *p* = 0.010), especially in low-risk group (HR = 1.56 (95%CI: 0.96–2.53), *p* = 0.07). In the multivariate analysis early recurrence was correlated with grade (OR = 2.47; 95% CI: 1.25–4.8; *p* = 0.008), pN (OR = 2.63; 95% CI: 1.55–4.54; *p* < 0.001), the number of lymph nodes harvested (OR = 0.51; 95% CI: 0.29–0.86; *p* = 0.013) and RDI-O (OR = 1.91; 95%CI: 1.06–3.39; *p* = 0.028). The early vs. late recurrence negatively correlated with OS regardless of the RDI-O (HR = 22.9 (95%CI: 13.9–37.6; *p* < 0.001).

**Conclusions:**

RDI-O < 60% in adjuvant therapy among stage III CC (especially in low-risk group) increases the risk of early recurrence within 18 months of surgery. Patients with early recurrence showed worse overall survival regardless of the RDI-O.

## Background

Significant advances have been made in the study of colon cancer (CC) in the last few years. In patients with stage III CC, oxaliplatin and fluoropyrimidine-based chemotherapy is currently the standard of therapy in the adjuvant setting [1–6]. Three prospective phase 3 trials: MOSAIC (Multicenter International Study of Oxaliplatin/5-Fluorouracil, Leucovorin in the Adjuvant Treatment of Colon Cancer), NASBP-C07 (National Surgical Breast and Bowel Project) and NO16968 (XELOXA Trial) showed improvements in prolonged disease-free time (DFS) and overall survival (OS), especially in younger patients under 65 years [1–6]. Similar efficacy, with better tolerability, has been demonstrated for capecitabine in the oxaliplatin regimen (CAPOX) [7]. The intended dose of oxaliplatin is usually reported as part of the design of clinical studies, but the administered dose is often reduced due to chemotherapy side effects such as myelotoxicity or peripheral neuropathy, which may increase the risk of early recurrence [1–6]. In colorectal cancer (CRC) patients, 60–80% of recurrences become apparent within the first 2 years of curative surgery and 90% within the first 4 years [8–10]. The factors affecting early and late recurrence in CRC may differ among stage and primary tumour sites [8, 10, 11]. The objectives of this study were to analyze the relationship between the relative dose intensity of oxaliplatin (RDI-O) in the adjuvant setting and early recurrence in stage III CC patients.

## Methods

### Study population

The study group included 365 stage III colon cancer patients of Caucasian race, age 18 or older, who underwent radical surgical treatment followed by adjuvant chemotherapy with fluoropyrimidine and oxaliplatin. The patients were diagnosed and treated between 2000 and 2014 in five oncology centers in Poland. The exclusion criteria included: rectal tumours (as defined by the presence of the inferior pole of the tumour below the peritoneal reflection (< 15 cm from the anal margin), unclear resection margins (residual tumour at the primary site: R1 or R2), inability to start adjuvant chemotherapy, and incomplete medical records. This study was approved by the Bioethics Committee of the Medical Chamber in Opole (Agreement No. 245/2017). All data were coded to secure full protection of personal information, therefore patient consent was not sought. All research was performed in accordance with relevant guidelines and regulations.

### Study treatment and procedures

The adjuvant chemotherapy included regimens with oxaliplatin and fluoropyrimidine FOLFOX-4 or CAPOX used for 6 months. For each regimen the number of cycles, the duration of treatment, the cumulative dose (CD) and RDI-O are given. The primary tumour staging was performed in accordance with the seventh version of the TNM classification, developed by the American Joint Committee on Cancer (AJCC) and International Union for the Fight against Cancer (IUCC) [12]. The assessment of the adverse events (AEs) severity was based on the classification developed by the National Cancer Institute (NCI-National Cancer Institute) - Terminology Criteria for Adverse Events (CTCAE), version 4 [13]. All AEs reported in medical records during the treatment period or within 30 days after the last chemotherapy cycle are listed. Safety analyses were done for patients who received at least one dose of treatment. Patients may have had more than one AE. The manuscript was prepared according to the STROBE guidelines [14]. Follow-up was measured from adjuvant chemotherapy initiation until death or the last follow-up information. DFS was defined as the period between chemotherapy initiation and disease recurrence (local or distant), while OS was defined as the period between chemotherapy initiation and death (irrespective of the cause). Thirty-six months was used as the cut off (< 36 vs ≥36) to define early and late recurrence. RDI represents the ratio of the amount of a drug actually administered to the amount planned for a fixed time period, according to formula: [DDI/SDI]× 100%. DDI (delivered dose intensity) is delivered total dose (in mg/m^2^)/standard time to complete chemotherapy (in days) and SDI (standard dose intensity) is standard total dose (in mg/m^2^)/actual time to complete chemotherapy. Imputation was used for missed cycles (in days) [15].

### Statistical design

The statistical analysis was performed using the R statistical software (version 4.0.0) [16]. The normality of distribution was assessed using the Shapiro-Wilk test. Due to the lack of normally distributed data, the comparison between the two groups was performed using the Mann-Whitney U test, while the comparison between multiple groups was performed using the Kruskal-Wallis test and the post-hoc Dunn test with the Benjamini-Hochberg correction for multiple testing [17]. The data were visualized with box-plots. The RDI-O cut-off was selected based on its association with DFS and OS. Survival analysis was performed using the Kaplan-Meier method [18]. Univariate analysis was performed using the Cox proportional hazard model; multivariate analysis was performed with the stepwise forward approach. Similarly, logistic regression analysis was performed for 12-month, 18-month and 36-month DFS, while the multivariate analysis employed the stepwise forward approach. For all analyses an α level of 0.05 was employed.

## Results

### Study population

The study group included 365 stage III colon cancer patients: 176 females (48.2%) and 189 males (51.8%) (Table 1). The mean age at diagnosis was 61.2 years (range 25–80 years), 176 (48.2%) patients were 63 years and 15.3% above 70 years. There were 176 patients (48.2%) with high risk (pT4 and/or pN2) and 189 (51.8%) with low risk (pT1–3 and pN1) stage III CC. The mean number of harvested lymph nodes was 13.2 (range 1–50). Left and right-sided primary tumours was 203 (55.6%) and 161 (44.1%), respectively. Most patients did not have diabetes at the time of diagnosis 325 (89%), and the median body mass index (BMI) was 26.4 (range 15.6–44). Other baseline clinical and pathology characteristics are summarized in Table 1.

**Table 1:** Patient characteristics

Variables	n 365 (100%)
Age at diagnosis; years	
Mean	62
Range	25–80
< 63	189 (51.8%)
≥ 63	176 (48.2%)
> 70	56 (15.3%)
Gender	
Female	176 (48.2%)
Male	189 (51.8%)
Body mass index (kg/m^2^)	
Median	26.4
Range	15 (6–44)
≤ 18,5	13 (3.6%)
18,5-24,9	128 (35.1%)
≥ 25,0	224 (61.4%)
Diabetes mellitus	
No	325 (89%)
Yes	40 (11%)
Histology	
Adenocarcinoma not otherwise specified (NOS)	304 (83.3%)
Mucinous adenocarcinoma	54 (14.8%)
Adenocarcinoma of cylindrical cells	2 (0.5%)
Signet ring adenocarcinoma	2 (0.5%)
Adenosquamous carcinoma	1 (0.3%)
Undifferentiated carcinoma	2 (0.5%)
Histopathology grade (G)	
G1 (well differentiated)	33 (9.0%)
G2 (moderate differentiated)	279 (76.4%)
G3 (poor differentiated)	52 (14.2%)
No data	1 (0.3%)
Primary tumor classification (pT)	
1	3 (0.8%)
2	47 (12.9%)
3	249 (68.2%)
4a	48 (13.2%)
4b	18 (4.9%)
Regional lymph nodes classification (pN)	
1a	106 (29.0%)
1b	119 (32.6%)
1c	1 (0.3%)
1	1 (0.3%)
2a	80 (21.9%)
2b	58 (15.9%)
Number of harvested lymph nodes	
< 12	196 (53.7%)
≥ 12	165 (45.2%)
No data	4 (1.1%)
Primary tumor location	
Ceacum	64 (17.5%)
Ascending colon	49 (13.4%)
Hepatic flexure	28 (7.7%)
Transverse colon	20 (5.5%)
Splenic flexure	22 (6.0%)
Descending colon	14 (3.8%)
Sigmoid colon	168 (46.1%)
Primary tumor location	
Right colon	161 (44.1%)
Left colon	203 (55.6%)
No data	1 (0.3%)
Surgery	
Right hemicolectomy	116 (31.8%)
Right hemicolectomy extended	36 (9.9%)
Left hemicolectomy	45 (12.3%)
Sigmoidectomy	167 (45.8%)
Transversectomy	1 (0.3%)
Adjuvant chemotherapy	
FOLFOX-4	336 (92%)
CAPOX (XELOX)	29 (8%)
CEA concentration before surgery; ng/ml	
< 5	96 (26.3%)
≥ 5	28 (7.7%)
No data	241 (66.0%)
CEA concentration after surgery; ng/ml	
< 5	292 (80.0%)
≥ 5	33 (9.0%)
No data	40 (11.0%)

### Treatment duration and safety

The median follow-up was 51.8 months (range 8.2–115.1). The majority of patients received FOLFOX-4 (336 patients; 92%) and 29 patients (8%) received CAPOX. The mean number of chemotherapy cycles was 10.2 (standard deviation 2.76). Two hundred and nine (57.3%), 45 (12.3%) and 31 (10%) patients received 12, 6 and < 6 cycles, respectively. The distribution of chemotherapy cycles was well balanced between clinical stages IIIA (pT_1–2_N_1a-c_/pT_1_N_2a_), IIIB (pT_3-4a_N_1a-c_/pT_2-3_N_2a_/pT_1–2_N_2b_), IIIC (pT_4a_N_2a_/pT_3-4a_N_2b_/pT_4b_N_1–2_), *p* = 0.47.

**Fig. 1:**
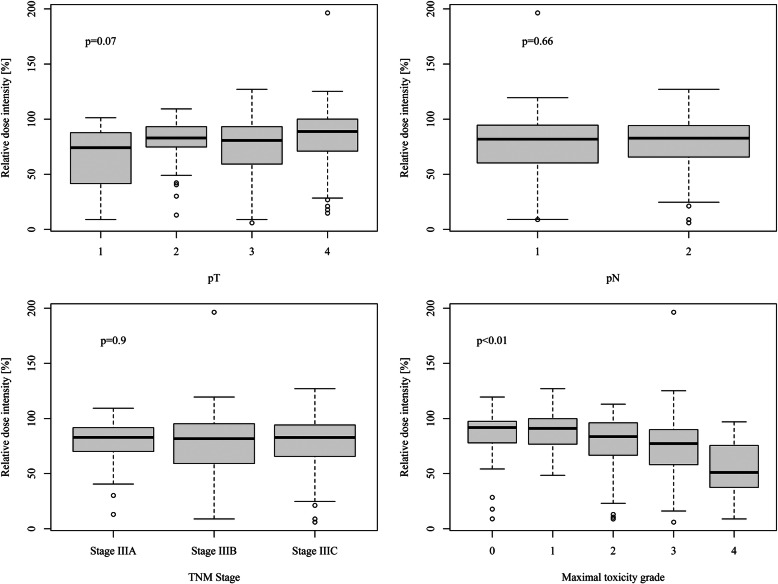
Relative dose intensity for oxaliplatin and pT, pN, TNM and maximal toxicity grade

The number of chemotherapy cycles was correlated with toxicity (Kruskal-Wallis-test *p* = 10^− 10^). Seventy-one patients (19.5%) discontinued oxaliplatin-based chemotherapy due to adverse events. The median cumulative dose (CD) and RDI-O were 936.86 mg/m^2^ (range 84.03–1042.70; interquartile range, IQR: 763.78–1016.94 [mg/m^2^]) and 82.32% (range 6.02–196.42, IQR: 61.37–94.27), respectively. In the group of patients who completed 12 chemotherapy cycles, CD was 1012.33 mg/m^2^ (range 632.50–1042.70; IQR: 961.50–961.50 [mg/m^2^]) and RDI-O was 91.58% (range 30.16–196.42, IQR: 83.01–98.94). There was no difference between median CD, RDI-O and pT (*p* = 0.46; *p* = 0.07), pN (*p* = 0.85; *p* = 0.66), pTNM stage (*p* = 0.92; *p* = 0.90) (Fig. 1). The CD and RDI-O were correlated with therapy toxicity (0 = 0.03 and *p* < 0.01). Oxaliplatin-induced peripheral neuropathy (OXIPN): sensory and motor occurred in 212 (58.1%), and 35 (9.6%) patients, respectively. Severe (grade 3) and life-threatening (grade 4) OXIPN were diagnosed in 54 (14.8%) and 9 (2.5%) patients. There was no relationship between diabetes and the risk of OXIPN (*p* = 0.31).

### Activity and efficacy

At the last follow-up 228 (62.5%) patients were alive and 137 (37.5%) had died. Median DFS was 43.86 months (range 0.79–113.42) and OS was 51.8 months (range 8.2–115.1). The number of cycles ≥6 vs. < 6 was not correlated with DFS and OS (HR = 0.68 (95% CI: 0.43–1.07) *p* = 0.09 and HR = 0.72 (95% CI: 0.43–1.18); *p* = 0.19, respectively.

Early recurrence within 36 months after surgery occurred in 130 patients (36.6%). The most common type of early relapse was distant metastases (100 patients; 76.9%); rarely local recurrence (8 patients; 6.2%) or both distant metastases and local recurrence (22 patients; 16.9%). The most common recurrence site was liver (74 patients; 60.7%), followed by the lung (41 patients; 33.6%), lymph nodes (27 patients; 22.1%), other organs (16 patients; 13.1%) and peritoneum (20 patients; 16.4%). Bone and brain metastases were diagnosed in 6 (4.9%) and 3 (2.5%) cases.

**Fig. 2:**
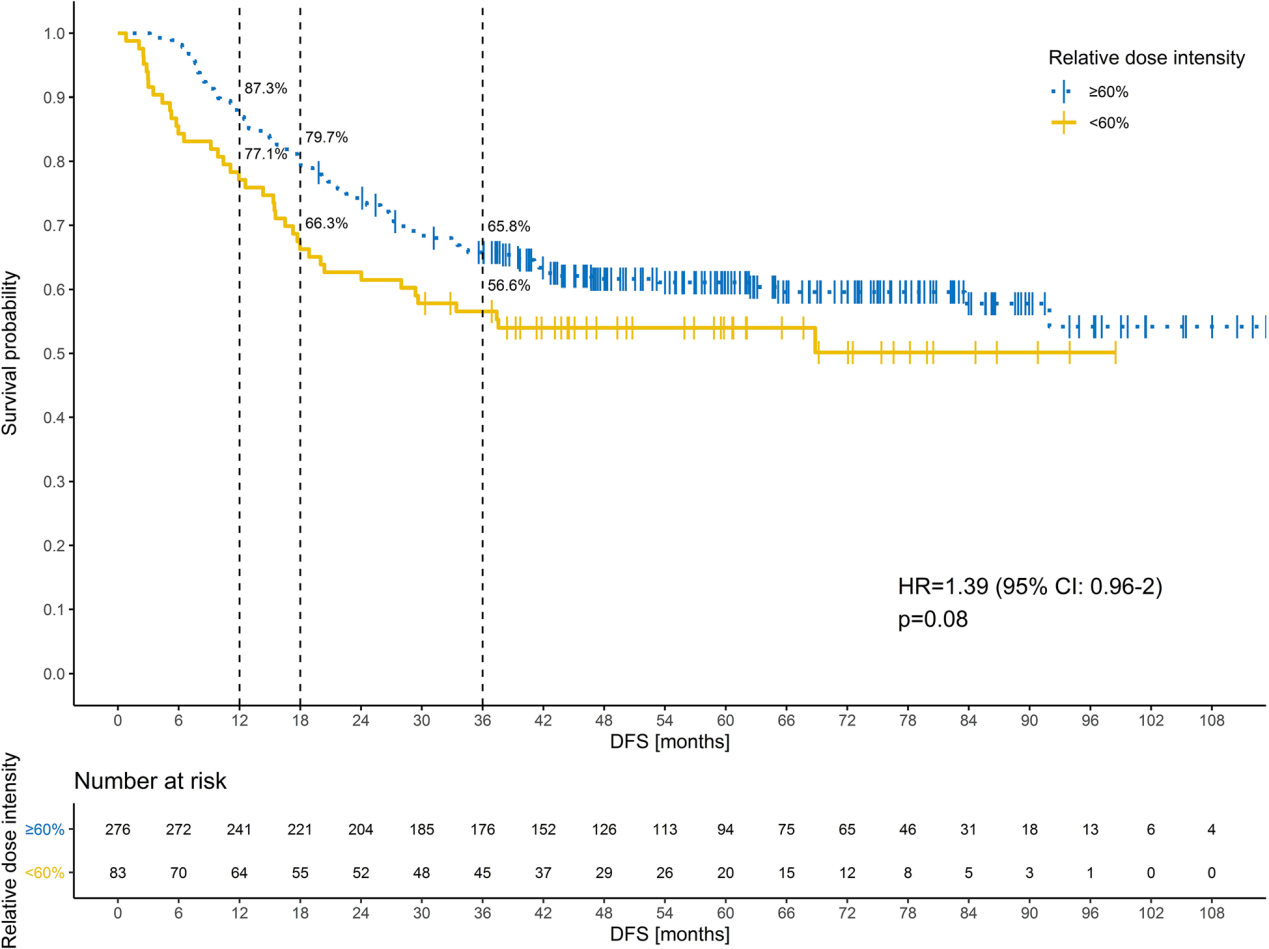
The relative dose intensity of oxaliplatin (RDI-O) ≥60 vs < 60% and early recurrence in the whole group within 12, 18 and 36 months

The RDI-O < 60% was related with recurrence within 12 and 18 months (OR = 2.04; 95%CI: 1.08–3.78; *p* = 0.024 and OR = 2.05; 95%CI: 1.18–3.51; *p* = 0.010) but not within 36 months (OR = 1.50; 95%CI: 0.90–2.48; *p* = 0.117) (Table 2), HR = 1.39 (95%CI: 0.96–2.0, *p* = 0.08 (Fig. 2).

**Table 2:** Uni- and multivariate logistic regression for disease recurrence within 12, 18, and 36 months

DFS	Variable	Grade 3 vs 2 vs 1	pT 4 vs 3 vs 2 vs 1	pN 2 vs 1	N^o^ harvested ≥ vs < 12	RDI-O < 60 vs ≥ 60%
		OR (95% CI); p				
12 months	Univariate analysis	2.82 (1.39–5.53); 0.003	3.03 (1.58–5.70); 0.001	1.78 (0.99–3.20); 0.051	0.59 (0.32–1.06); 0.078	2.04 (1.08–3.78); 0.024
	Multivariate analysis	2.36 (1.09–4.93); 0.025	2.84 (1.40–5.65); 0.003	1.93 (1.03–3.63); 0.041	0.49 (0.25–0.92); 0.028	2.06 (1.03–4.01); 0.036
18 months	Univariate analysis	2.45 (1.30–4.55); 0.005	1.93 (1.07–3.68); 0.027	2.23 (1.36–3.68); 0.002	0.61 (0.37–1.00); 0.051	2.05 (1.18–3.51); 0.010
	Multivariate analysis	2.47 (1.25–4.80); 0.008	–	2.63 (1.55–4.54); < 0.001	0.51 (0.29–0.86); 0.013	1.91 (1.06–3.39); 0.028
36 months	Univariate analysis	2.09 (1.15–3.80); 0.015	2.80 (1.62–4.88); < 0.001	2.17 (1.39–3.39); 0.001	0.62 (0.40–0.96); 0.034	1.50 (0.90–2.48); 0.117
	Multivariate analysis	1.93 (1.02–3.65); 0.043	2.66 (1.49–4.79); 0.001	2.46 (1.53–4.01); < 0.001	0.46 (0.28–0.74); 0.001	–

*DFS* Disease free survival, *CI* Confidence interval, *OR* Odds ratio, *p* Pathological, *N*^*o*^ Number, *RDI-O* Relative dose intensity of oxaliplatin

**Fig. 3:**
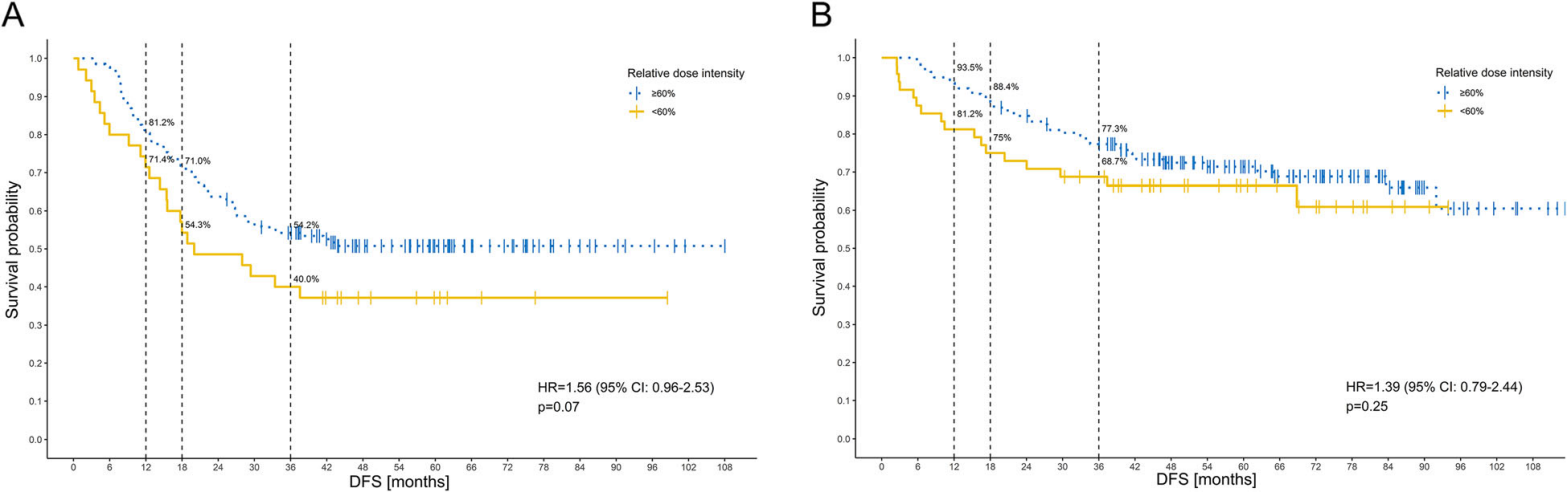
The relative dose intensity of oxaliplatin (RDI-O) ≥60 vs < 60% and early recurrence in low-risk (**a**) and high-risk (**b**) subgroups within 12, 18 and 36 months

The low-risk group recurrence within 12 and 18 months (OR = 3.31; 95%CI: 1.21–9.04; *p* = 0.018 and OR = 2.73; 95%CI: 1.16–6.37; *p* = 0.020) and high-risk group (OR = 1.72; 95%CI: 0.71–3.96; *p* = 0.209 and OR = 2.06; 95%CI: 0.96–4.42; *p* = 0.062) (Table 2); HR = 1.56 (95%CI: 0.96–2.53), *p* = 0.07 (Fig. 3a) and HR = 1.39 (95%CI: 0.79–2.44; *p* = 0.25) (Fig. 3b).

In the univariate analysis, other factors which correlated with recurrence within 12 and 18 months were tumor grade (OR = 2.82; 95%CI: 1.39–5.53; *p* = 0.003 and OR = 2.45; 95%CI: 1.30–4.55; *p* = 0.005), pT (OR = 3.03; 95% CI: 1.58–5.70; *p* = 0.001 and OR = 1.93; 95% CI: 1.07–3.68; *p* = 0.027), pN (OR = 1.78; 95% CI: 0.99–3.20; *p* = 0.051 and OR = 2.23; 95% CI: 1.36–3.68; *p* = 0.002) (Table 2). In the multivariate analysis, recurrence within 12 and 18 months correlated with grade (OR = 2.36; 95%CI: 1.09–4.9; *p* = 0.025 and OR = 2.47; 95% CI: 1.25–4.8; *p* = 0.008), pT (OR = 2.84; 95% CI: 1.4–5.65; *p* = 0.003), pN (OR = 1.93; 95% CI: 1.03–3.63; *p* = 0.041 and OR = 2.63; 95% CI: 1.55–4.54; *p* < 0.001), the number of lymph nodes harvested (OR = 0.49; 95% CI: 0.25–0.92; *p* = 0.028 and OR = 0.51; 95% CI: 0.29–0.86; *p* = 0.013) and RDI-O (OR = 2.06; 95%CI: 1.03–4.01; *p* = 0.036 and OR = 1.91; 95%CI: 1.06–3.39; *p* = 0.028) (Table 2).

In the multivariate analysis, recurrence within 36 months correlated with grade (OR = 1.93; 95%CI: 1.02–3.65; *p* = 0.043), pT (OR = 2.66; 95%CI: 1.49–4.79; *p* = 0.001), pN (OR; 95% CI: 1.53–4.01; *p* < 0.001), and the number of lymph nodes harvested (OR = 0.46; 95%CI: 0.28–0.74; *p* = 0.001) (Table 2). The early vs. late recurrence negatively correlated with OS regardless of the RDI-O (HR = 22.9 (95%CI: 13.9–37.6; *p* < 0.001).

## Discussion

The postoperative, oxaliplatin-based adjuvant chemotherapy for stage III CC patients demonstrated an improvement in patient outcome and has become the standard of care. In clinical trials, FOLFOX/CAPOX in adjuvant setting has been shown to cause a statistically significant improvement in DFS and OS over fluorouracil-based chemotherapy (4–7% and 2–6%, respectively) [1–6]. Further, in the MOSAIC and NO16968 study an improvement in OS was detected after a longer, approximately seven-year, follow-up [2, 6].

Unfortunately, adverse events caused by oxaliplatin often lead to premature termination of therapy and thus a reduction in the number of cycles or dose, and consequently the CD and RDI [2–6]. In the MOSAIC, NASBP-C07 and NO16968 trials, approximately 30% of patients receiving oxaliplatin did not complete the planned treatment due to adverse events, mainly burdensome OXIPN [1–3]. Similarly, in our study about 20 % of patients discontinued treatment, mainly due to OXIPN which occurred in various grades in 212 patients (58.1%). Therefore, the number of adjuvant oxaliplatin-based chemotherapy cycles that may be enough in this group of patients remains an open question. Tsai et al. have shown that at least eight FOLFOX cycles are needed to have OS benefit, and seven to ensure DFS [19]. Moreover, in the International Duration Evaluation of Adjuvant Therapy (IDEA) project in stage III CC patients, the non-inferiority of FOLFOX/CAPOX regimens used for 3 vs. 6 months was not demonstrated [20–22]. However, in the lower risk group (pT1–3/N1), the 3-month (4 cycles) CAPOX was as effective as the 6-month (8 cycles) treatment and the 3-year rate of DFS was 74.6 and 75.5%, respectively [20–22]. Importantly, the shorter treatment was associated with a lower risk of adverse events, including OXIPN.

To the best of our knowledge, the impact of RDI-O in adjuvant therapy among stage III colon cancer patients on early recurrence has not been systematically addressed, either in a prospective or a retrospective fashion. The correlation between the RDI of systemic therapy and clinical outcomes has been demonstrated mainly for diffuse lymphoma [23–25] and metastatic solid tumours that are relatively sensitive to anti-tumour drugs [26–28]. In stage III CC, a retrospective study of 367 patients treated with fluoropyrimidine-based chemotherapy mainly without oxaliplatin between 2003 and 2008 at 19 VA medical centers in the USA showed an RDI of chemotherapy above 70%, improving 5-year OS [29]. It should be noted that in this study RDI was calculated for each drug within each regimen: i.e. 5-fluorouracil, capecitabine and leucovorin [29]. One study directly evaluated the prognostic impact of oxaliplatin dose reduction in the adjuvant setting, but in stage II and III colorectal cancer [30]. In this study of South Korean patients it was observed that more than 60% of standard dose of oxaliplatin should be administered to achieve no difference in 5-year DFS and OS [30].

In our study of homogenous Caucasian stage III CC patients treated mainly with FOLFOX-4 (*N* = 336, 92%), no relationship was found between numbers of cycles: ≤6 vs. > 6 cycles and DFS or OS. However, it should be observed that the majority of patients received more than 6 cycles of chemotherapy (*N* = 320, 88%). Due to the oxaliplatin dose reduction in subsequent chemotherapy cycles, the median CD-O was 936.86 mg/m^2^, and in the group of patients who completed 12 cycles, 1012.33 mg/m^2^. However, the CD-O in our group treated in clinical practice was higher compared to the doses in the MOSAIC and NASBP-C07 studies (respectively 810 and 677 mg/m^2^) but the clinical outcomes were slightly worse, with a 3-year DFS 61.64% and median DFS 43,86 months [1–3]. The results in patients participating in clinical trials are generally better than in patients treated in everyday practice for a variety of reasons. However in our study, half of patients met the criteria of low-risk of relapse (189, 51.8%) but only 165 patients (45.2%) in the whole cohort had at least twelve lymph nodes removed during surgery. The small number of examined nodes may be “under-staged” and affect the prognosis. However, some reports suggest that the total number of lymph nodes analyzed in stage III CC is not a prognostic indicator of cancer-specific and DFS [31, 32].

Among CC patients, 80% of recurrences become apparent within the first 3 years and an additional 15% between the 3rd and 5th year of curative surgery [8–10, 33, 34]. Previous studies showed that stage III CC patients were more prevalent in the early-recurrence group than in the late-recurrence group, and had worse clinical outcomes [8, 10, 11, 35]. In our study, the factors associated with early recurrence (within 18 months) were tumour grade, the number of positive and harvested lymph nodes, and RDI-O < 60%. Interestingly, the risk of early recurrence in patients with RDI-O < 60% concerned the low-risk group in particular. These results should be referred to the aforementioned IDEA project and CAPOX efficacy in low-risk group [20–22]. It seems that RDI-O may be a more accurate reflection of the true patient-relevant benefit of adjuvant chemotherapy among stage III CC. However, patients with early recurrence showed worse overall survival regardless of the RDI-O.

We are aware that the retrospective nature of the study may have influenced our findings. Moreover, in our study we focused only on RDI-O not each drug within regimen and the risk of early recurrence. Further, we did not collect data on molecular abnormalities in primary tumour, e.g. the presence of activating mutations in the *KRAS, NRAS* and *BRAF*, or microsatellite instability and defective DNA mismatch repair (dMMR). However, previous research suggests that dMMR seems to be important only among stage II patients being considered for single-agent, fluoropyrimidine-based therapy [36]. Further research should focus on better biomarkers to assess the likelihood of chemotherapy response, based on molecular biology and pharmacokinetic analyses to reduce toxicity and improve treatment outcomes.

## Conclusions

We have demonstrated that RDI-O under 60% in adjuvant setting among stage III CC patients apparently increases the likelihood of early recurrence, especially in the low-risk group. It should be noted that research into the optimal dose of oxaliplatin in adjuvant treatment is important, due to the lack of effective methods of prevention and therapy of long-term OXIPN, which negatively affects patients’ quality of life [37].

## Data Availability

The dataset used and analyzed in this study are not publicly available due to the data generated for the reporting but available only to the research members on reasonable request.

## Abbreviations

AEs: Adverse events
AJCC: American Joint Committee on Cancer
BMI: Body mass index
BRAF: Proto-oncogene, serine/threonine kinase
CAPOX/XELOX: Chemotherapy schedule (oxaliplatin, capecitabin, leucovorin)
CC: Colon cancer
CD: Cumulative dose
CEA: Carcinoembryonic antigen
CRC: Colorectal cancer
CTCAE: Common Terminology Criteria for Adverse Events
DDI: Delivered dose intensity
DFS: Disease-free survival
dMMR: Defective DNA mismatch repair
FOLFOX: Chemotherapy schedule (oxaliplatin, 5-fluorouracil, leucovorin)
HR: Hazard ratio
IDEA: International Duration Evaluation of Adjuvant Chemotherapy collaboration
IQR: Interquartile range
IUCC: International Union for the Fight against Cancer
KRAS: Kristen rat sarcoma viral oncogene homolog
MOSAIC: Multicenter International Study of oxaliplatin/5-fluorocil, Leucovorin in Adjuvant Treatment of Colon Cancer
NASBP-C07: National surgical Breast and Bowel Project
NCI: National Cancer Institute
NRAS: Neuroblastoma RAS viral oncogene homolog
OS: Overall survival
OXIPN: Oxaliplatin-induced peripheral neuropathy
R1: Microscopic tumour infiltrations
R2: Macroscopic tumour infiltrations
RDI: Relative dose intensity
RDI-O: Relative dose intensity of oxaliplatin
SDI: Standard dose intensity
STROBE: Strengthening the Reporting of Observational Studies in Epidemiology
TNM: TNM classification of Malignant Tumours
